# Effectiveness of a mindfulness-oriented substance use prevention program for boys with mild to borderline intellectual disabilities: study protocol for a randomised controlled trial

**DOI:** 10.1186/s12889-020-09878-w

**Published:** 2020-11-25

**Authors:** Lucie Waedel, Anne Daubmann, Antonia Zapf, Olaf Reis

**Affiliations:** 1 Department of Child and Adolescent Psychiatry and Neurology, University Medical Centre Rostock, Gehlsheimer Str. 20, 18147 Rostock, Germany; 2 Department of Medical Biometry and Epidemiology, University Medical Centre Hamburg-Eppendorf, Martinistr. 52, 20246 Hamburg, Germany

**Keywords:** Mild to borderline intellectual disability, Alcohol, Prevention, Adolescents, Mindfulness, Substance use

## Abstract

**Background:**

Boys with mild to borderline intellectual disabilities (MBID) are at particular risk to drink in harmful ways once they start to consume alcohol. Interventions based on mindfulness have been proven to be effective in preventing substance use, but mostly for adults with MBID. A mindfulness oriented intervention targeting 11–17 years old boys will be tested in a randomised controlled trial. Study aim is to investigate the benefits of this new intervention compared to an active control condition within a 12 months follow-up.

**Methods:**

In this randomised controlled proof of concept study, 82 boys with MBID who consumed any alcohol during the last year will be randomised either to the 6 week mindfulness oriented intervention or the control group receiving a control intervention equal in dose and length. The intervention group undergoes mindfulness training combined with interactive drug education, while the control group completes a health training combined with the same education. In the intention-to-treat analysis the primary outcome is the self-reported delay of first post-intervention drunkeness within a 12 months follow-up time span, measured weekly with a short app-based questionnaire. Secondary outcome is the use of alcohol, tobacco and other drugs within 30 days post-intervention. Changes in neurobiological behavioural parameters, such as impulse control, reward anticipation, and decision making, are also investigated. Other secondary outcomes regard trait mindfulness, emotion regulation, psychopathological symptoms, peer networks, perceived stress, and quality of life. In addition, a prospective registry will be established to record specific data on the population of 11–17 year old boys with MBID without any alcohol experience.

**Discussion:**

This study offers the opportunity to gain first evidence of the effectiveness of a mindfulness-oriented program for the prevention of substance use for boys with MBID.

**Trial registration:**

German Clinical Trials Register, DRKS00014042. Registered on March 19th 2018.

## Background

### Background and rationale

Across many nations, the prevalence of alcohol consumption rises steadily from early adolescence to adulthood. As for underage drinking, a recent U.S. National Survey on Drug Use and Health (NSDUH) reported about 29.8% of 15-year-olds who had drunk alcohol once in their life [1]. According to the European School Survey Project on Alcohol and Other Drugs (ESPAD), almost half (47%) of 15–16-year olds reported drinking alcohol at the age of 13 or younger [2]. In numerous rankings, Germany appears as one of the countries where adolescents drink high quantities of alcohol [3]. According to data from the study on Health Behavior of School Children 2017/18 in Germany (HBSC), 8.7% of 11-year-olds reported alcohol consumption at least once in their lives. This number goes up to one third (32.1%) for 13-year-olds and three quarters (72.4%) for 15-year-olds [4]. Similar increases were observed for smoking [5]. In another German study conducted by the Federal Centre for Health Education in 2018, approximately 4600 children and adolescents were surveyed, with about 1% of 14–15-year-olds reporting consumption of risky quantities. This percentage rises to almost 10% among 16–17-year-olds [6], given that 10 g of pure alcohol consumption for women (one standard drink) and more than 20 g for men (two standard drinks) are considered as risky [7]. About 14% of 12–17-year-olds reorted at least 1 day of binge drinking in the previous 30 days (five drinks per occasion for men/ four drinks for woman) [6]. While, historically, lifetime and 30-day prevalence of alcohol consumption decreased significantly between 1995 and 2015, heavy episodic alcohol consumption stayed about this level [2]. Several studies indicate that early substance use predicts substance use later in life [8, 9]. In a German prospective longitudinal study, Laucht and Schmid observed that three quarters of 15-year-olds who had tried alcohol were also alcohol consumers later in life, one-fifth of them being weekly drinkers [10].

Learning to consume alcohol in a non-harming way constitutes an important developmental task of adolescence. Young people with limited intellectual abilities face even greater developmental challenges [11]. In a recent review, Van Duijvenbode & VanDerNagel examined 138 studies on substance use among subjects with mild to borderline intellectual disabilities (MBID, IQ 50–85). For people with MBID aged between 11 and 21 years, the lifetime prevalence of alcohol consumption ranged between 15.6 and 75.4% [12]. A study by Emerson compared drinking behaviours of adolescents with and without MBID and found lower prevalence for adolescents with MBID (41%) compared to those without (50%) [13]. This result resonates with our own study where we found higher prevalences of adolescents without MBID drinking (79.9%) when compared to adolescents with MBID (63.5%) [11]. In our study, however, we found the proportion of abstainers to be higher in pupils with MBID, which leaves a greater proportion of adolescents who are yet about to start drinking. However, a study by Pacoricona et al. found no significant differences between both groups [14]. Another study by Emerson reported slightly higher prevalence for adolescents with MBID (15.8%), compared to 13.2% in those without MBID [15]. Contradictory results of studies comparing adolescents with and without MBID may result from methodological weaknesses, as many studies do not control for confounding factors, such drinking habits of the parents or parental education [11].

According to the 2018 NSDUH study, the lifetime prevalence of any alcohol use disorder (AUD) is about 1.6% in 12–17 year olds of the general population in the U.S. [16]. However, in adolescents diagnosed with a MBID the lifetime prevalence rates for substance use disorders (SUD) seem to be higher as they range between 0.1–2.7% [12]. Along with our findings, authors of the review conclude that adolescents with MBID compared to age mates without MBID have a higher risk of subsequent problems, once they start consuming [11, 12]. In our study in special schools, boys with MBID, in particular, showed a significantly higher risk of consuming alcohol in a more harmful way and of becoming intoxicated once they start drinking. They also ran into conflict with the law more often and more quickly compared to their age-mates without MBID or to girls with MBID [11]. The duration between the onset of drinking and the first drunkenness was about 11 months for students without MBID and about half the time for students with MBID. Risk factors for alcohol consumption in this group were male sex [11, 17], smoking [15], having friends who use substances [15, 18], and the degree of cognitive impairment [12]. Boys with MBID constitute a special group since in contrast to boys with more severe impairments they have a more independent lifestyle, are more closely involved in social life, and therefore participate more in the “normal” cultural life giving them better access to substances. With increased participation, conflicts associated with social comparison and negative role models become more likely. More severe behavioural and emotional problems, such as anxiety, aggression, depression or exposure to prejudice, are assumed to be additional predictors of substance use for this group [19, 20]. Confounding factors, such as lower socio-economic status, unfavourable living conditions, low activity lifestyles, difficulties in getting in touch with peers, and negative role models are reported to be more frequent for this group [12].

Intending to address a special target group, we developed a prevention program based on mindfulness, a modern approach to intensify all sorts of treatment, which we combine with interactive education about addictions. It aims at the high-risk group of male adolescents with MBID, who have started drinking alcohol.

The concept of mindfulness has its historical roots 2500 years ago in the religious context of Asia [21]. Coming from this historical context, the former Zen student and professor emeritus of the University of Massachusetts Medical School, Jon Kabat-Zinn, is regarded as the pioneer who transferred mindfulness into the medical-scientific context. In 1979 he founded a clinic for stress reduction where he taught his Mindfulness Based Stressed Reduction program (MBSR) and undertook the first systematic examination [22]. Since then, mindfulness has been increasingly adopted as a psychological and therapeutic element in healthcare to reduce stress, pain and illness. Publications on the subject have grown exponentially during the last 20 years [23, 24]. In most cases, mindfulness is defined as a moment-by-moment experience with clear-eyed attention to the working of the mind, body and behaviour [25, 26]. The regulation and maintenance of focused attention and awareness lead to an experience of wakefulness called state mindfulness [27, 28]. Mindfulness can also be conceptualised as a stable trait assumed to be a part of the personality, which is pronounced differently in individuals and can be increased by regular practice [28]. Mindfulness means not only perceiving the current moment, but also having a non-judgmental attitude towards it. For instance, mindfulness-based approaches expand behavioural therapy programs by promoting the development of acceptance and the ability to distinguish between changeable and unchangeable conditions in order to manage and perceive everyday life in a more self-determined way. In the treatment of addiction, there is promising evidence from general populations that mindfulness-based interventions can reduce alcohol consumption, general craving and the cue-induced craving for alcohol [29, 30]. Higher trait-mindfulness is associated with lower substance use [31]. According to the meta-analysis of Dunning et al. on children and adolescents, mindfulness is positively associated with self-control, executive functioning and attention, and is inversely associated with depression, anxiety/stress and negative social behaviour [32].

With the help of mindfulness, we want to strengthen individual resources for conscious decision making. We assume that the drug education program enriched by mindfulness also influences psychological and behavioural moderators of substance use. Addiction prevention programs in schools have proven to be effective when they use interactive teaching methods [33]; therefore we use interactive methods in drug education that deal with different addictions and addictive behaviour in a playful and task-oriented manner. This concept will be proven in a randomised controlled trial.

### Specific objectives of the study

The study aims to provide initial evidence that mindfulness can play an important role in preventing boys with MBID from consuming alcohol, tobacco and other drugs (ATOD), with alcohol being the focus of interest. Furthermore, we like to target and evaluate the role of bio-behavioural factors in improving addiction-related outcomes. The effectiveness of the intervention is evaluated in a randomised controlled trial with an active control condition and weekly follow-ups over a period of 12 months to test the following hypotheses: (1) The intervention is more effective than the active control condition with regard to time between the end of the training and the first event of drunkenness within 1 year after treatment (primary outcome). More precisely, the time span between treatment and first drunkenness event should be longer for the intervention group when compared to the control group. (2) Participants from the intervention group show stronger decrease in ATOD consumption (secondary outcome). (3) The effectiveness of the intervention is associated with changes in the neurobiological parameters of self-regulation, changes in mindfulness, psychopathological symptoms (strengths and weaknesses), and changes in perceived stress and health-related quality of life. Other parameters like reward anticipation, decision making, and impulse control will be examined on an exploratory basis (for details see Statistical analysis section below).

## Methods/design

### Study setting

The study takes place in the city of Rostock, a city of 200.000 inhabitants in the German federal state of Mecklenburg-Western Pomerania. Boys from special schools for children with learning disabilities and/or with mild to borderline intellectual disabilities will be invited to participate in the study. Moreover, boys meeting the inclusion criteria will be recruited from out- and inpatient psychiatric units and other relevant institutions. A continuing list of cooperating centres for recruitment will be established with the registry.

### Trial design

**Fig. 1:**
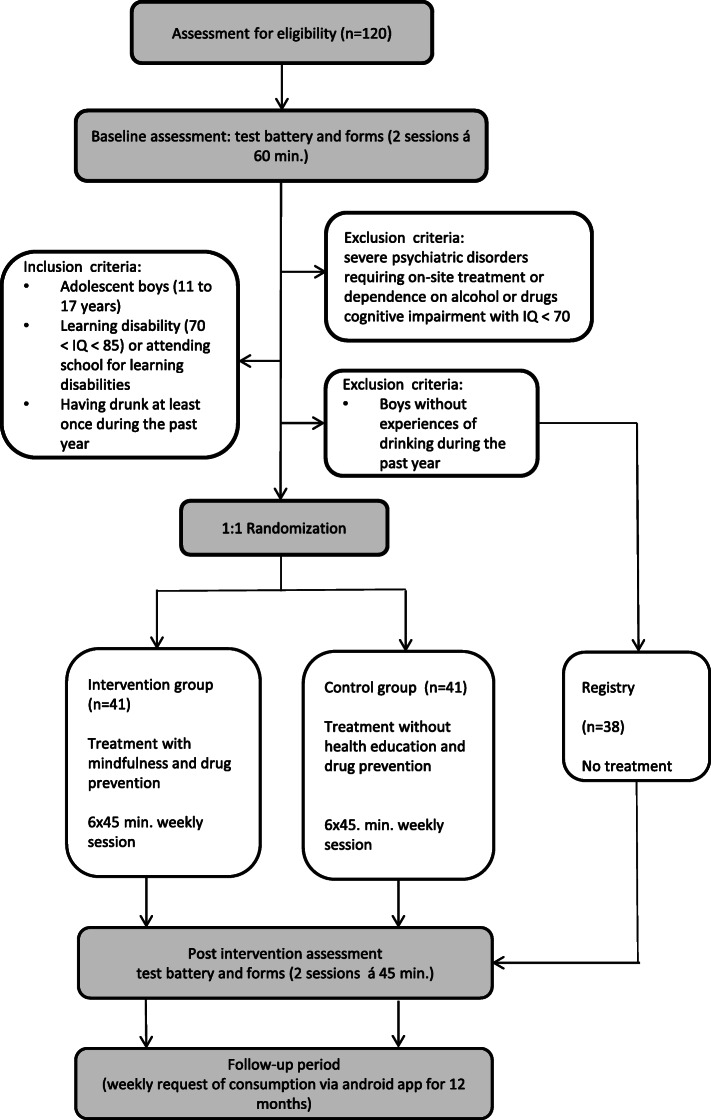
Study flow diagram

The study evaluates the effectiveness of a mindfulness-based intervention in preventing ATOD use in 11–17 years old boys with MBID with special focus on the consumption of alcohol. All adolescents fulfilling inclusion criteria will be randomised to either the intervention group or the active control condition (see Fig. 1). Persons who do not have experiences with alcohol will be invited to be part of a registry. With the registry data, we describe probabilities of starting to consume alcohol depending on personal risk factors. The study is designed as a prospective, randomised, controlled superiority trial proving our concept with two parallel groups. This randomised controlled trial (RCT) will be combined with a small add-on registry study. The first study arm comprises a mindfulness-oriented intervention, the second a sham intervention equal in length and dose, but different in content. Participants from both groups join a 6-week program conducted in one-to-one sessions. The intervention group receives a training containing mindfulness exercises and elements of drug prevention. The control group receives a training that contains exercises in health education instead of mindfulness. Participants in the registry receive no intervention and are measured at the same intervals and using the same measurement methods. The trial involves two assessment points (pre- and post-intervention) and a 12 months follow-up period with weekly measurements via the android app movisensXS starting at the last intervention session. Figure 1 shows the flow diagram of the current trial design.

### Procedure

The study timeline is shown in Table 1. Boys who have consumed any alcohol within the last 12 months before initial assessment and who and their parents/caregivers have given written informed consent will be included in the study (t_0_ timing of enrolment). They undergo the baseline measures about the length of two sessions (t_1_ and t_2_). In case they do not fulfill the inclusion criteria of alcohol use they will be invited to be part of the non-intervention registry. In case they fulfill all inclusion criteria participants will be randomised to either the intervention group or control group in a 1:1 allocation ratio. A central randomisation list will be created with variable block lengths stratified by the recruitment locations school vs. clinics, outpatient clinics and practices. Enrolled participants will be randomized on the base of computer-generated random numbers provided by a research associate of the Institute of Medical Biometry and Epidemiology (IMBE) of the University Medical Center Hamburg-Eppendorf. This assistant is not involved in the practical implementation of the research project. After randomisation, participants get the training over six sessions. After completion of the treatment, participants take part in the post-measurements (t_3_-t_4_). During the post-measurement, the app (movisensXS) is installed on the smartphone of the participants. In case of compatibility problems, a smartphone is handed out to the test persons by the study staff. This smartphone will be only usable for the follow-up survey. For 12 months after intervention, every Sunday afternoon, participants get an app-based reminder and are asked to join the short follow-up survey. Here, they retrospectively report on their substance consumption for the last week directly on their smartphones (t_5_-t_57_) by answering questions via the app.

**Table 1:**
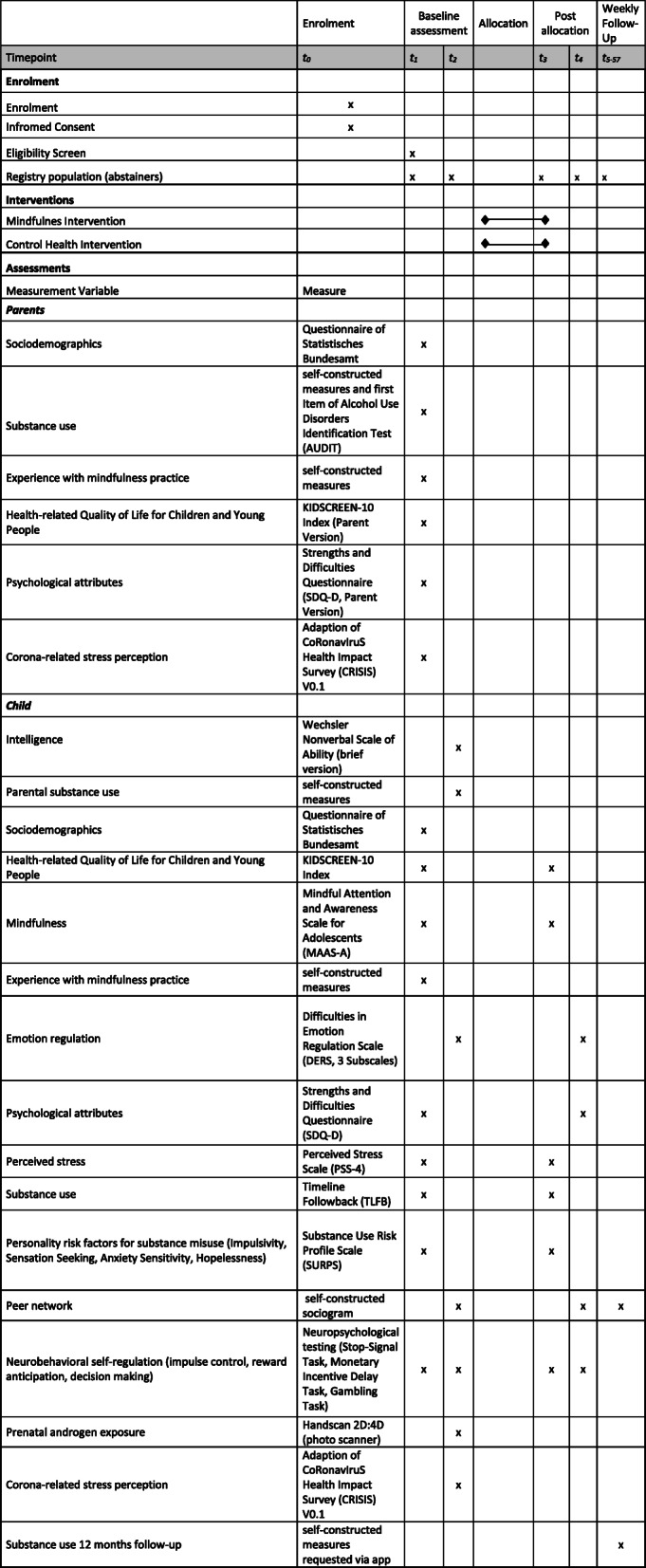
Participant timeline of enrolment, treatment and measurement

A voucher worth 5€ from a sales brand of a leading consumer electronics retailer can be earned for each training or measurement session.

### Intervention

#### Mindfulness-oriented substance use prevention program

The intervention results from an extensive research of the literature, a focus-group with teachers from a school for children with learning disabilities, and a feasibility study on *n* = 32 participants. In an individual face-to-face setting the participants will receive six 45 min sessions once a week. The training is conducted by a person experienced in applications of mindfulness.

The mindfulness practice contains classic elements of Mindfulness Based Stress Reduction (MBSR) and mindfulness exercises adapted for children and youth. The six lessons involve an introduction to mindfulness and exercises on various themes: Awareness of the present moment, body awareness, observation of thoughts and cognition, stress management, perceiving and dealing with positive and negative emotions, and issues that may have an influence on substance use, such as trigger cues and interacting with peers. As basic sources we used “Using the Wisdom of Your Body and Mind to Face Stress, Pain, and Illness: How to Cope with Stress, Pain and Illness” by Kabat-Zinn [25, 34], “Mindfulness-Based Substance Abuse Treatment For Adolescents” by Himmelstein & Saul [35], “Mindfulness Curriculum for Adolescents by Mindful Schools” [36], “Sitting Still Like a Frog: Mindfulness Exercises for Kids” by Snel [37] and “Learning to Breathe” by Broderick [38].

Sessions of the intervention also include elements of drug education. This subject will be addressed with interactive exercises to meet the requirements of young people with MBID. These tasks are about different addictions, the ability to recognise problematic behaviour, and reflections on stress and resilience factors.

All exercises were tested during an 18-month feasibility period for practicability, comprehensibility and simplicity of tasks to perform, as well as for cognitive load, simple language and length. All sessions, however, are planned for providing sufficient space for explanation and repetition. As the training was extensively piloted, no other modifications during the proof of concept phase are planned.

Since sessions need to fit into school schedules, sessions were adapted to the time frame of a school lesson. In school, sessions are held between or after school hours in a room provided by the school. In the case of outpatient treatment, the sessions take place at the participants’ homes, on the premises of the study centre, or in rooms provided by cooperating institutions.

All participants are performing small home works after each training session for the time between the weekly sessions. These are fun tasks to remind them of mindfulness and invite them to try it out in practice and integrate techniques and knowledge into their everyday lives.

#### Control condition: health education

There is evidence that mindfulness is a promising treatment approach for people with intellectual disabilities, e.g. in the reduction of aggression [39–41]. However, in a review by Chapman et al. [42] on the effectiveness of mindfulness-based interventions in people with intellectual disabilities, these investigations were criticised for being subject to methodological shortcomings. For example, not the treatment but the attachment to or the interpersonal skills of the trainer would be the reason for pre-post changes [42]. In order to control the effect of social bonding, the control group takes place under an active condition. Participants receive an intervention that equals the non-control intervention in terms of setting (face-to-face), dose and duration but, deals with “hot” health issues. Participants will explore three major themes of healthy food, sports and sexuality, with each topic extending over two sessions. As in the mindfulness-condition, content is delivered via interactive tasks, small games, assignment tasks and movement exercises. Both treatments are structured and manualised to the same degree [43] and elements of drug education are equal for both groups (see Table 2). As in the mindfulness-condition, after each session participants will receive fun tasks as homework for weekly reminders of the contents.

**Table 2:** Training manuals for intervention group and control group

Session	Intervention group: Mindfulness Intervention	Control group: Health Intervention
Session 1	Welcome and overview, flashlight for mood (every session), meaning of mindfulness, attitudes of mindfulness, first meditation practice, introduction of the breath as an anchor	Welcome and introduction, flashlight for mood, (every session) Healthy food: Nutrition pyramid, food ingredients; Nutri-Score rating system and reflection of the own eating behaviour
Session 2	Meditation practice, mindful coping with desire, mindful eating a raisin vs. piece of chocolate	Healthy food: Hidden sugar in your food, game with sugar cubes, taking pictures of packaged food with the amount of sugar
Session 3	Be mindful, sense your body, introduction to and practice of the body scan + Desire, craving and consumption, self-rating of consumption	Meaning of sports and physical health in everyday life, current body posture, ergonomic sitting tools + Desire, craving and consumption, self-rating of consumption
Session 4	Meditation practice, moods and current emotions, drawing moods as a weather report + Memory Game: Addiction and addictive behaviour	Development, long term effects and consequences of bad motion habits, sport exercises for a healthy and strong back + Memory Game: Addiction and addictive behaviour
Session 5	Mindfulness of emotion: Identify positive, negative and neutral emotions, stop the autopilot, sense emotions + Card game: “Enjoying life”	Sex education: Reconsideration of gender stereotypes; sexuality in the context of pleasure, love and responsibility + Card game: “Enjoying life”
Session 6	Mindfulness of thoughts: The power of thoughts, observe and produce thoughts, mindful walking + Card game: Stress in different situations, Evaluation and personalised stress management	Sex education: sexual harassment in online networks and chats, strategies for action to say no + Card game: Stress in different situations, Evaluation and personalised stress management

#### Inclusion and exclusion criteria

Boys will be included in the randomised trial if

- they and their parents/cargegivers give informed consent
- they have declared that they have drunk alcohol during the past year
- they are between 11 and 17 years (clinical/institution recruitment) or between 13 and 17 years (school recruitment)
- they are diagnosed with having a MBID (70 < I.Q. < 85) or if they are educated in a school for learning disability

The decision about fulfilling the inclusion criteria, and therefore randomisation, can be made only after informed consent was given. This is due to the fact, that many boys have problems to admit, that they drank alcohol, when their parents are around. A clear picture on their consumption pattern will be gained only in the more intimate situation of the first measurement (t_1_). Interested boys who meet the criteria but who had not drank alcohol in the previous year are directed to the registry (see Fig. 1) and receive neither the intervention nor the control intervention. Based on estimations from the feasibility study, we plan an oversampling of at least *n* = 38 persons.

Boys will be excluded from randomisation if they have 1) no experiences of drinking in the last year; 2) display severe psychiatric disorders requiring on-site treatment or dependence on alcohol or drugs; 3) have a cognitive impairment with I.Q. ≤ 70. In case they fulfill the second or third criteria, boys will be excluded completely from the study. Boys only fulfilling the first criterion will be directed into the registry and will be observed for a possible initiation of drinking during the study period of 1 year.

### Data collection

#### Primary outcome

Events of drunkenness are collected retrospectively after completion of the training on a weekly basis by the movisensXS app for the last 7 days. From these measurements, the time to first event of drunkenness is derived. In case of drunkenness events, quantitative measures of alcohol consumption are taken as numbers of units (bottles, glasses of various beverages) in an easy-to-answer manner. A weekly electronic measurement at 5 pm every Sunday proved to be feasible as did the research tool designed by the researchers on a flexible platform (movisensXS, Version 1.5.8, (movisens GmbH, Karlsruhe, Germany)).

#### Secondary and other outcomes

Secondary outcomes include self-reported alcohol use during the first year after intervention as a quantitative measure of consumption taken on a weekly basis using a smartphone-based research tool.

For all participants, the number of cigarettes will be inquired weekly.

Furthermore, emotion regulation, neurobehavioural self-regulation, trait mindfulness, psychological attributes, perceived stress and health-related quality of life and will be measured before and after the intervention. In case of drunkenness events, the weekday of the event and event-related social networks will be recorded. The consumption of illicit drugs of any kind will be asked for on a weekly basis. Sociodemographic data on the adolescent’s family contain consumption habits in the parental household (specific target variables and measurements are listed in Table 1).

### Plans for data collection

#### Follow up

##### Substance use

Our sampling method via movisensXS involves asking participants weekly about drunkenness events and the consumption of alcohol, tobacco and illicit drugs. For binging events, they will be asked for the timing, quantity and experience. The questioning takes a maximum of 3 min. It starts the first week after the intervention and should be carried out weekly for a total of 52 weeks.

#### Pre/ post measurements

Most of the measures taken are harmonised throughout the projects of the IMAC-consortium [44–47].

##### Sociodemographics

Items are based on the questionnaire of the Statistisches Bundesamt for recording sociostructural survey characteristics in the population [48].

##### Substance use

The Timeline Followback (TLFB) is a calendar method in which participants are asked retrospectively about their consumption of alcohol, nicotine, cannabis and other drugs, establishing a 30-days-before baseline and a post-measurement calendar [49].

##### Family risk factors

In order to record risky alcohol consumption, addiction or abuse, the alcohol consumption of parents/caregivers is measured with the help of a German translation of the 10-item Alcohol Use Disorders Identification Test (AUDIT) [50], of which one item will be used. Health-relevant data on smoking, sport, eating and sleeping behaviour in the family will be collected via a self-authored items.

##### Mindfulness

The Mindful Attention Awareness Scale-Adolescents (MAAS-A) for young people between 14 and 18 years of age contains 15 items for the assessment of dispositional mindfulness [51]. The questionnaire takes a maximum of 10 min with a good internal consistency (alpha = .82) and retest reliability (r = .79).

##### Personality

Substance Use Risk Profile Scale (SURPS) is a 23-item self-assessment questionnaire that includes four personality risk factors for substance abuse (impulsiveness, sensation-seeking, anxiety sensitivity, and hopelessness) [52].

##### Intelligence

The Wechsler Nonverbal Scale of Ability (WNV) is a non-verbal general cognitive ability assessment for children, teenagers, and young adults aged 4 to 21. The brief version lasts requires 15–20 min and consists of the two subtests matrices and spatial span [53]. It will be used in cases when no sufficient information about intelligence level or schooling can be found.

##### Neuropsychological testing

This neuropsychological computer-based testing includes three separate test paradigms.

1. Stop-Signal Task for recording response inhibition.
2. Monetary Incentive Delay Task measuring reward anticipation.
3. Cambridge Gambling Task assessing decision-making and risk-taking behaviour.

The neuropsychological test battery takes about 40 min and was designed by a subproject of the IMAC consortium [54].

##### Perceived stress

The short form of the Perceived Stress Scale (PSS-4) is a four-item well-validated self-assessment of perceived stress during the last month [55].

##### Emotion regulation

The Difficulties in Emotion Regulation Scale (DERS; Gratz & Roemer, [56], German version Ehring et al., [57]) is a well-validated and reliable self-report measure of emotion regulation and dysregulation (Cronbachs alpha = .95, split-half reliability = .96) targeting the 11–17 year olds. In the current trial, we are focusing on 3 of the 5 subscales, (1) Difficulties engaging in goal-directed behaviour (goals), (2) Impulse control difficulties (impulse) and (3) Limited access to emotion regulation strategies (strategies).

##### Health-related quality of life

The health-related quality of life of children and adolescents will be assessed with the Kidscreen-10 Index by means of self-disclosure and external assessment by parents in a pre measurment [58]. The 10-item-questionnaire provides a good internal consistency reliability (Cronbach’s Alpha = .82) and a moderate retest reliability (r = .73).

##### Influence of peers

A small self-developed network inventory asks for peer influences on alcohol and tobacco consumption. Three name-generators of most important friends are explored in greater detail for descriptive and injunctive social contagion, selectivity, and importance.

##### Psychopathological symptoms

The German version of the Strengths and Difficulties Questionnaire (SDQ, [59]; German version by Woerner [60, 61], is suitable for children and adolescents between 3 and 17 years of age. The test comprises 25 positive and negative attributes of the 5 subscales emotional symptoms, conduct problems, hyperactivity/inattention, peer relationship problems and prosocial behaviour. The same 25 items are included in a questionnaire version to be filled in by the parents.

##### Impact of the Covid-19 situation

Corona-related stress perception is measured with a short adaptation of CoRonavIruS Health Impact Survey (CRISIS) V0.1 [62]. The questionnaire consists of 12 items in the parent version and 9 items in the children version.

##### Prenatal androgen exposure

During the intrauterine period, androgen exposure organises the brain with lifelong neurobiological and behavioural effects. There is multi-level evidence that higher prenatal androgen exposure increases the risk for substance use disorders in later life [63–66] and also affects mindfulness [67] and the body mass index [68]. Within the projects of the IMAC-Mind Consortium, the participants’ second-to-fourth-finger length ratios (2D:4D), a proxy of prenatal androgen exposure, will be quantified via optical measurement.

### Statistical analysis

#### Sample size

For the primary outcome “time to first event of drunkenness”, we calculated the sample size for a logrank test comparing the intervention and control group. In the literature, we did not find data referring to students with MBID for precise estimating of the base rates of survival. Hence, we refer to the European School Survey Project on Alcohol and other Drugs (ESPAD, see Introduction). ESPAD-data indicates that about 60% of students report at least one event of drunkenness during the last year, which makes an event-free survival rate of 0.4 in the control group. In the absence of evidence-based assumptions on the effectiveness of the intervention, we estimate that the intervention will drop down the events of drunkenness from a base rate of 60 to 28%, which corresponds to an event-free survival rate of 0.72 in the intervention group. This leads to a hazard ratio of 0.36. With a power of 80% and a significance level of 5% (two-sided hypothesis), we need an overall sample size of 82 participants (41 participants per group) to detect this difference. We assume that we lose 30% of the participants in each group during the study duration. These observations are censored. Furthermore, we expect no one to switch groups. The study is planned without accrual period.

For the most important secondary outcomes “quantity of alcohol units /number of tobacco cigarettes”, we can show median effects of .65 in terms of Cohens d with this sample size and with a type I error of α = .05 (two-sided hypothesis), a power of 1-β = .80 and a correlation between baseline and follow-up of r = .50. The sample size calculation was conducted with PASS 15 based on the modules “Logrank Tests” and “Analysis of Covariance”.

#### Statistical methods for primary and secondary outcomes

The descriptive statistics will be presented separately for every group and for the total sample. The primary and secondary analysis will be based on the intention-to-treat population, which includes all participants randomised. For the primary outcome “time to first event of drunkenness”, a logrank test will be calculated to compare group differences in survival curves. The resulting statistical test for group comparison will be performed two-sided at the 5% significance level. The analysis will be repeated in the per-protocol population. In an additional analysis, we will extend this time-to-event-analysis to a Cox regression to adjust our results for the following covariates: intelligence, age, mindfulness, mental health, and family risks. If the event “drunkenness” occurs frequently enough in the individual participant, for the secondary analysis we will use an appropriate extension of the Cox model to deal with recurrent events and a Poisson regression using the count of the events as the dependent variable. For the most important secondary outcomes, change from baseline of the number of alcohol units and change from baseline of the number of cigarettes, a baseline-adjusted linear mixed model will be calculated for each outcome with participants as a random effect and group (intervention vs. control), time and recruitment location (schools vs. clinics, outpatient clinics, and practices) as fixed effects, and the respective baseline value as a covariate. The time by group interaction will be tested, and if the interaction is not significant, the interaction will not be included in the model. In this model, missing values will be directly imputed to enable an intention-to-treat analysis, which results in unbiased estimators under the missing-at-random-assumption. For further secondary outcomes (neuropsychological testing and questionnaires), a baseline adjusted analysis of covariance will be determined with the respective change from baseline to post-intervention as a dependent variable, group and recruitment location as independent variables, and the respective baseline value as a covariate. The analysis of all secondary outcomes will be performed in an exploratory manner. Interim analyses are not planned. Exploratory analyses will be performed for possible intervening factors, such as neurobehavioural parameters, mindfulness (trait) or quality of life. The analysis of the registry study will be performed in an exploratory manner. Probabilities of starting to consume alcohol will be described descriptively.

Standard statistical software as STATA (Version 16.0 or newer), R (Version 4.0.3 or newer), SAS (Version 9.4 or newer) or SPSS (Version 25 or newer) will be used for the statistical analyses.

## Discussion

Although there are several mindfulness programs for children and adolescents, we found no programs aimed at adolescents with MBID. With this study, we want to test a new program tailored to this group. A previously conducted feasibility study showed that mindfulness can be successfully applied and implemented in children and adolescents with MBID. In a randomised controlled study, we want to test the effectiveness of the newly developed program against an active control condition to prove this concept.

The prevention program combines elements based on currently published and partly evaluated mindfulness programs and courses designated for the use in children and adolescents, based on the work of Jon Kabat-Zinn. Since we cannot exclude the possibility that an intensive engagement with the young people alone will foster all sorts of improvements in mindfulness and health behaviour, we have chosen an active control condition, which is carried out in a similar face-to-face way and is identical in terms of dose and length to the intervention. Both the intervention and the control program are combined with elements of classical drug education which were adapted in comprehensibility and difficulty to youth with MBID. For the implementation of the program, we have consciously decided on a face-to-face setting as it was used for people with reduced cognitive abilities [69]. That way, we are able to better meet the individual needs of the boys with MBID, but also to exclude other interfering social factors, such as social pressures to play “cool”. With our study, we want to evaluate whether mindfulness can help substantially to reduce alcohol consumption among young boys with MBID. Based on the secondary outcomes, we plan to assess whether mindfulness has further effects on neurobehavioural regulation, experiences and the everyday life of the adolescents. If the concept proves to be effective, we would published the results in peer-reviewed journals and mindfulness could be seen as a new promising approach in prevention work with young people with MBID.

### Trial status

Protocol version number v 1.0, 31.07.2020. The study has now begun and recruitments starts in November 2020.

## Data Availability

Not applicable.

## Abbreviations

ATOD: Alcohol, tobacco and other drugs
AUD: Alcohol use disorder
AUDIT: Alcohol Use Disorders Identification Test
CRISIS: Coronavirus Health Impact Survey
DERS: Difficulties in Emotion Regulation Scale
DLR: Deutsches Zentrum für Luft- und Raumfahrt e. V [German Aerospace Center]
ESPAD: European School Survey Project on Alcohol and Other Drugs
HBSC: Health Behaviour of School Children
IMAC-Mind: Improving Mental Health and Reducing Addiction in Childhood and Adolescence through Mindfulness
IMBE: Institute of Medical Biometry and Epidemiology
MBID: Mild to borderline intellectual disabilities
MBSR: Mindfulness-based stress reduction
MASS-A: Mindful Attention Awareness Scale for Adolescents
MTF: Monitoring the Future study
NSDUH: National Survey on Drug Use and Health
PSS: Perceived Stress Scale
RCT: Randomised controlled trial
SDQ: Strengths and Difficulties Questionnaire
SUD: Substance use disorder
SURPS: Substance Use Risk Profile Scale
WNV: Wechsler Nonverbal Scale of Ability (WNV)
